# Can temperament dimensions predict treatment outcome in inpatients with substance use disorders?

**DOI:** 10.1192/j.eurpsy.2025.639

**Published:** 2025-08-26

**Authors:** E. Santens, E. Dierckx, G. Dom, L. Claes

**Affiliations:** 1 Addiction Psychiatry, Alexian Psychiatric Hospital, Tienen; 2 Faculty of Medicine and Health Sciences, University of Antwerp, Antwerp; 3 Developmental and Life Span Psychology, Vrije Universiteit Brussel, Brussels; 4 Alexian Psychiatric Hospital, Tienen; 5 Faculty of Psychology and Educational Science, KU Leuven, Leuven, Belgium

## Abstract

**Introduction:**

Substance use disorders are among the leading causes of morbidity and mortality worldwide. SUDs are highly comorbid with other mental health disorders. Given this comorbidity, a transdiagnostic view on treatment, seems appropriate. Within such a transdiagnostic perspective, treatment outcome can be described as a decrease in comorbid clinical symptomatology and not merely in terms of abstinence/relapse in substance use. A promising transdiagnostic factor within the RDoC framework is temperament, more specifically reactive and regulative temperament. According to the dual pathways model, psychopathology arises from an imbalance between two complementary neurobiological systems: the bottom-up reactivity system in terms of behavioral inhibition (BIS) and behavioral activation (BAS) (reactive temperament) and the top-down regulation in terms of Effortful Control (EC) (regulative temperament).

**Objectives:**

We want to investigate whether reactive (BIS/BAS) and regulative temperament (EC) are associated with treatment outcome in terms of a decrease in clinical symptomatology in a sample of adult inpatients with a SUD. When these temperamental factors turn out to be significant predictors of clinical symptomatology, treatment interventions targeting reactivity (high BAS or BIS level) or aiming at strengthening EC could possibly result in better treatment outcomes for patients with SUDs and comorbid disorders.

**Methods:**

The sample consisted of 612 inpatients with a SUD ((76,5% males, mean age 42,9 years) admitted at a specialized treatment unit for addiction. At the start of the treatment (pre) self-report questionnaires were administered to assess the reactive temperament dimensions (the Behavioral Inhibition/Behavioral Activation System Scales), the regulative temperament dimension (the Effortful Control Scale from the Adult Temperament Questionnaire) and clinical symptomatology (Symptom-Checklist-90-Revised, SCL-90-R). At discharge, the SCL-90-R was administered again to assess treatment effectiveness (post).

**Results:**

Paired sample t-test showed significant decreases between pre- and posttreatment symptom scores indicating that treatment was effective in decreasing symptomatology. A hierarchical regression analysis showed that higher levels of EC were associated with a stronger decrease in levels of psychological symptoms and that higher levels of BIS were associated with a lower decrease. There was however no moderating role of EC in the relation between reactive temperamental dimensions and treatment outcome.

**Conclusions:**

We found that reactive and regulative temperament could predict psychological symptomatology after a residential treatment period of 8 weeks in a specialized addiction unit. These results point out that interventions aiming at either strengthening EC or lowering anxiety (BIS) could possibly result in better treatment outcomes for patients with SUDs their comorbid disorders.

**Disclosure of Interest:**

None Declared

